# Retraction: MiR-519b-3p inhibits the proliferation and invasion in colorectal cancer via modulating the uMtCK/Wnt signaling pathway

**DOI:** 10.3389/fphar.2023.1285746

**Published:** 2023-09-06

**Authors:** Frontiers Editorial Office

The journal retracts the 02 July 2019 article cited above.

Following publication, concerns were raised regarding the integrity of the images in the published figures. The authors failed to provide the raw data or a satisfactory explanation during the investigation, which was conducted in accordance with Frontiers’ policies. Given the concerns about the validity of the data, and the lack of raw data, the editors no longer have confidence in the findings presented in the article.

This retraction was approved by the Chief Editors of Frontiers in Pharmacology and the Chief Executive Editor of Frontiers. The authors agree with this retraction.

